# SARS-CoV-2 antibodies in inflammatory neurological conditions: a multicentre retrospective comparative study

**DOI:** 10.1007/s12026-023-09384-2

**Published:** 2023-05-12

**Authors:** Cecilia Zivelonghi, Alessandro Dinoto, Sarosh R. Irani, Andrew McKeon, Andrea Pilotto, Alessandro Padovani, Stefano Masciocchi, Eugenio Magni, Chiara R. Mancinelli, Ruggero Capra, Giorgia T. Maniscalco, Irene Volonghi, Ava Easton, Daniela Alberti, Gianluigi Zanusso, Salvatore Monaco, Gian Luca Salvagno, Giuseppe Lippi, Sergio Ferrari, Sara Mariotto

**Affiliations:** 1 Department of Neurosciences, Biomedicine and Movement Sciences, Neurology Unit, University of Verona, Verona, Italy; 2 Oxford Autoimmune Neurology Group, Nuffield Department of Clinical Neurosciences, University of Oxford, Oxford, UK; 3 Department of Neurology, John Radcliffe Hospital, Oxford University Hospitals, Oxford, UK; 4 Oxford Epilepsy Research Group, University of Oxford, Oxford, UK; 5 Department of Laboratory Medicine and Pathology, Rochester, MN USA; 6 Department of Neurology Mayo Clinic, Rochester, MN USA; 7 Department of Clinical and Experimental Sciences, Neurology Unit, University of Brescia, Brescia, Italy; 8 Neurology Unit, Poliambulanza Hospital, Brescia, Brescia, Italy; 9 Multiple Sclerosis Center, ASST - Spedali Civili of Brescia, Brescia, Montichiari Italy; 10 Multiple Sclerosis Center “A. Cardarelli” Hospital, Naples, Italy; 11 Neurological Clinic and Stroke Unit “A. Cardarelli” Hospital, Naples, Italy; 12 Encephalitis Society, 32 Castlegate, Malton, UK; 13 Department of Clinical Infection, Microbiology and Immunology, University of Liverpool , Liverpool, England; 14 Section of Clinical Biochemistry, University of Verona, Verona, Italy; 15 Service of Laboratory Medicine, Pederzoli Hospital, Peschiera del Garda, Verona, Italy

**Keywords:** SARS-CoV-2, Encephalitis, Demyelinating disorders, Viral trigger

## Abstract

It is well established that neurological and non-neurological autoimmune disorders can be triggered by viral infections. It remains unclear whether SARS-CoV-2 infection induces similar conditions and whether they show a distinctive phenotype. We retrospectively identified patients with acute inflammatory CNS conditions referred to our laboratory for antibody testing during the pandemic (March 1 to August 31, 2020). We screened SARS-COV-2 IgA/IgG in all sera by ELISA and confirmed the positivity with additional assays. Clinical and paraclinical data of SARS-COV-2-IgG seropositive patients were compared to those of seronegative cases matched for clinical phenotype, geographical zone, and timeframe. SARS-CoV-2-IgG positivity was detected in 16/339 (4%) sera, with paired CSF positivity in 3/16. 5 of these patients had atypical demyelinating disorders and 11 autoimmune encephalitis syndromes. 9/16 patients had a previous history of SARS-CoV-2 infection and 6 of them were symptomatic. In comparison with 32 consecutive seronegative controls, SARS-CoV-2-IgG-positive patients were older, frequently presented with encephalopathy, had lower rates of CSF pleocytosis and other neurological autoantibodies, and were less likely to receive immunotherapy. When SARS-CoV-2 seropositive versus seronegative cases with demyelinating disorders were compared no differences were seen. Whereas seropositive encephalitis patients less commonly showed increased CSF cells and protein, our data suggest that an antecedent symptomatic or asymptomatic SARS-CoV-2 infection can be detected in patients with autoimmune neurological conditions. These cases are rare, usually do not have specific neuroglial antibodies.

**Supplementary Information:**

The online version contains supplementary material available at 10.1007/s12026-023-09384-2.

## Introduction

SARS-CoV-2 primarily causes a respiratory infection, but accumulating case reports and few isolated multicentre studies have described patients with concomitant CNS disorders [1–3]. Indeed, a large self-controlled case study has demonstrated an increased incidence of demyelinating events, myelitis, encephalitis, and meningitis following SARS-CoV-2 positivity [4]. Most of these larger studies are observational and lack appropriate control groups. Given many neurological conditions are idiopathic, these populations carry great importance, particularly in a pandemic scenario. Despite this, systematic comparisons of patients with and without concomitant or antecedent SARS-CoV-2 infection and CNS symptoms remain unreplicated.

Herein, we assess SARS-CoV-2 seropositivity in a consecutive cohort of patients referred for neurological autoantibody testing to explore the prevalence of SARS-CoV-2-IgG positivity and ask whether clinical and paraclinical features differed between seropositive and seronegative patients with suspected CNS autoimmune disorders.

## Methods

### Study subjects and patients

We retrospectively identified patients referred to the Laboratory of Neuropathology, University Hospital of Verona, Italy, for testing of autoantibodies against myelin oligodendrocyte glycoprotein (MOG), aquaporin-4 (AQP4), and onconeural or neuronal cell surface antigens between March 1, 2020 and August 31, 2020. Of the 391 consecutive patient samples, we excluded 39 samples of patients with a chronic disease or referred during a relapse of a known condition and 13 patients in whom only CSF was available. From the remaining 339 cases, 232 were referred for autoantibody analyses related to demyelinating diseases and 107 to autoimmune encephalitis.

### SARS-CoV-2 antibody testing

SARS-COV-2 IgA and IgG were analyzed in all sera with a S1 spike protein subunit based FDA-approved ELISA assay (Euroimmun, Germany). Available CSF from SARS-CoV-2-IgG seropositive patients were also tested, as previously described [5]. Positive results were validated using a trimeric anti-SARS-CoV-2 S1/S2 IgG test (DiaSorin, Saluggia, Italy), an anti-SARS-CoV-2 receptor binding domain (RBD) IgG test (Beckman-Coulter, Brea, USA), and an anti-SARS-CoV-2 RBD total antibodies test (Roche Diagnostics, Basel, Switzerland). Patients were defined as SARS-CoV-2-IgG positive if at least one of the confirmatory tests and the screening test were positive.

### Study subjects and controls

SARS-CoV-2-IgG-positive patients were classified according to their clinical phenotype at hospital discharge into two groups: (1) “suspected autoimmune encephalitis/encephalopathy” which included patients with autoimmune encephalitis or encephalopathy and new-onset refractory status epilepticus -NORSE; and (2) “atypical demyelinating disorders” including isolated optic neuritis, isolated myelitis, acute disseminated encephalomyelitis (ADEM), neuromyelitis optica spectrum disorder—NMOSD-, and MOG-IgG-associated disorder—MOGAD. Encephalitis, limbic encephalitis, and ADEM were defined according to diagnostic criteria [6, 7]. As controls, we collected information from SARS-CoV-2 IgA/IgG seronegative patients who had a matched clinical phenotype and enrolled in the same timeframe and from the same centres of SARS-CoV-2-positive patients. The selection of seronegative cases from the same geographical area and in the same timeframe is meant to avoid any possible bias related to the presence of different SARS-CoV-2 strains. Similarly, the selection of matched clinical phenotypes is meant to avoid biases related to the inclusion of cases referred for autoantibody testing despite a low pre-test probability of being positive (i.e. multiple sclerosis in the atypical demyelinating disorders group). Lastly, clinical information from patients referred from other institutions were anonymously collected by referring physicians in an electronic spreadsheet.

### Autoantibody testing

A live cell-based immunofluorescence assay was used to analyse antibodies to MOG [8], a fixed cell-based assay to detect autoantibodies against neuronal cell surface antigens and aquaporin-4, and a line blot followed by immunohistochemistry on rat cerebellum to test and confirm autoantibodies to intracellular/synaptic antigens (Euroimmun, Germany).

### Statistical analysis

Differences in categorical variables were assessed with Chi-square test or Fisher Test, as appropriate. Mann–Whitney test was applied to compare median values. *P* value < 0.05 was considered statistically significant.

### Standard protocol approvals, registrations, and patient consents

This study was in accordance with the ethical standards of the institutional and national research committee and with the 1964 Helsinki Declaration, and its later amendments or comparable ethical standards, and was approved by the local Bioethics Committee (Comitato Etico per la Sperimentazione Clinica, Azienda Ospedaliera Universitaria Integrata di Verona; BIOB-NEU-DNA-2014, protocol 13,582).

## Results

We identified SARS-CoV-2 IgA and/or IgG in 23/339 (7%) patients: IgA and IgG in 13, IgA only in 9 and IgG only in 1. After additional SARS-CoV-2 assays, 16 of 23 (16/339; 4%) patients were confirmed as seropositive cases; the remaining 7 patients with SARS-CoV-2 IgA positivity only at screening assay were excluded. From CSF testing, 4 patients were positive for SARS-CoV-2 IgG at the screening assay, with 3/4 confirmed on additional testing.

**Fig. 1:**
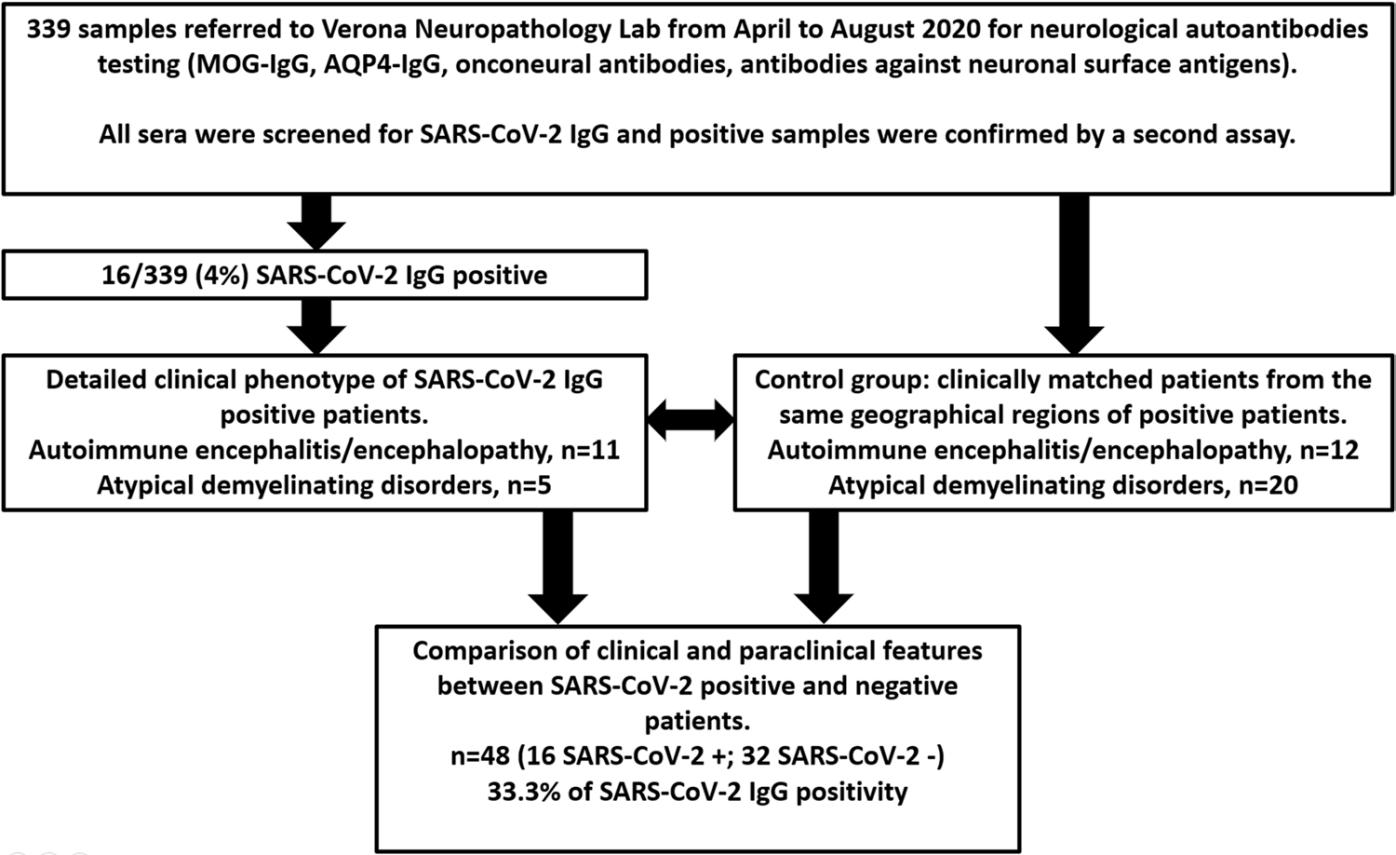
Flowchart of the study design

**Fig. 2:**
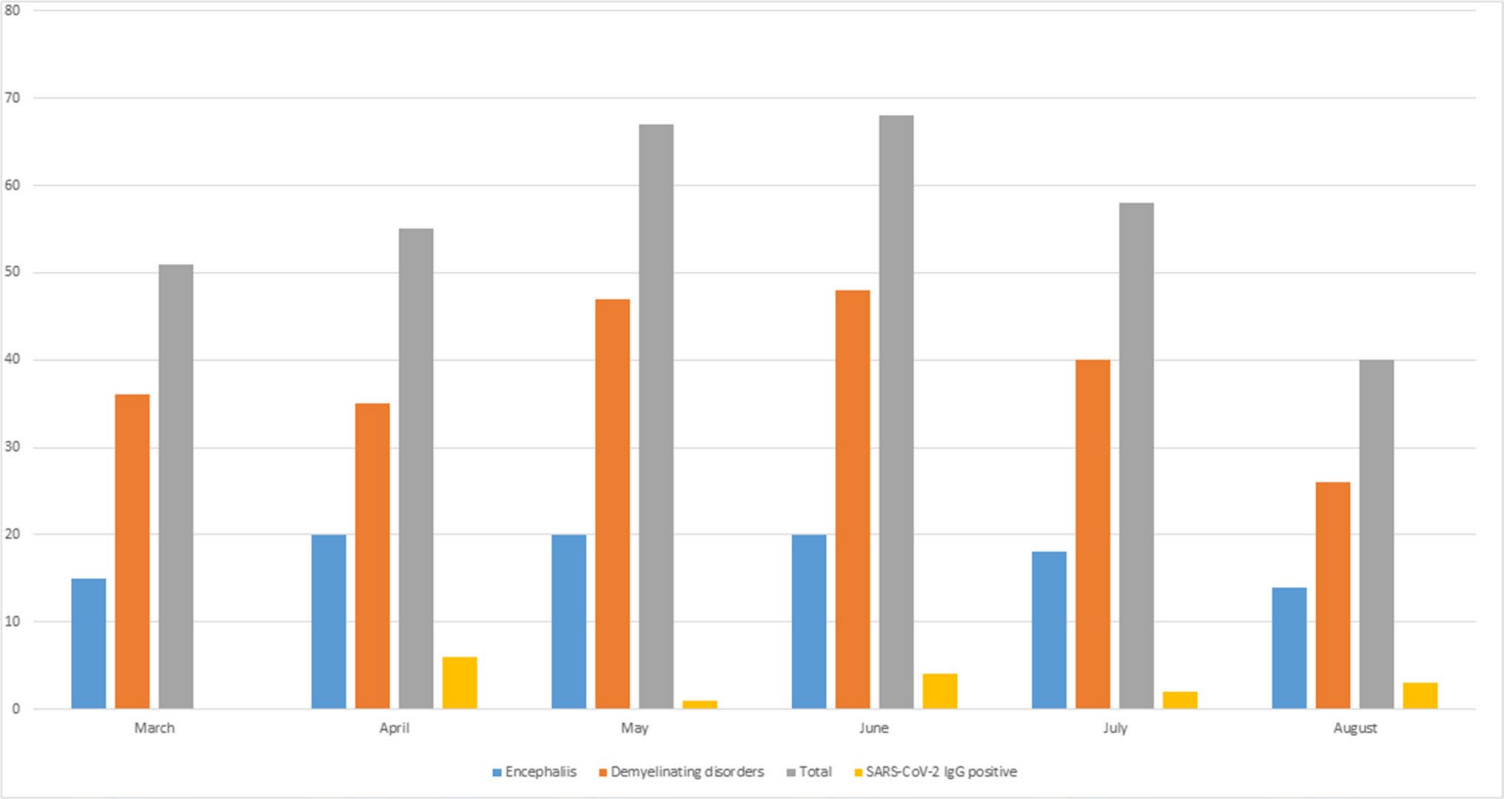
Monthly data of samples referred for antibody testing (total, grey, and divided according to the clinical phenotype of demyelinating disorders, orange, and encephalitis, light blue) in relation to the number SARS-CoV-2 IgG-positive patients (yellow) per month

As controls, we included all (*n* = 32) consecutive SARS-CoV-2-IgG seronegative patients with matched clinical features who were referred from the same geographical areas in the same timeframe (Fig. 1). Over the 6 months of study, SARS-CoV-2 IgG positivity and neurological autoantibodies showed similar rates (Fig. 2).

Overall, from the 48 seropositive and seronegative cases (summarized in Table 1 and Fig. 2), 23 were in the “suspected autoimmune encephalitis/encephalopathy” cohort (11 seropositive, 12 seronegative) and 25 in the “atypical demyelinating disorders” cohort (5 seropositive, 20 seronegative).

**Table 1:** Clinical features of included patients regardless of SARS-CoV-2 serostatus (*n* = 48)

Age	54 (5–83)
Female gender	29/48 (60.4%)
Significant comorbidities	15/48 (31.3%)
Seizures	12/48 (25%)
Myelitis	8/48 (16.7%)
Optic neuritis	13/48 (27.1%)
Encephalopathy	25/48 (52.1%)
Abnormal MRI	34/44 (70.8%)
Abnormal EEG	19/19 (100%)
CSF pleocytosis	24/44 (54.5%)
CSF cells	7.5 (0–299)
CSF increased protein concentration	21/44 (47.7%)
CSF protein concentration, mg/dL	42.65 (17–4395)
CSF restricted oligoclonal bands	8/33 (24.2%)
Neurological autoantibodies positivity	12/48 (25%) *
SARS-CoV-2 antibodies positivity	16 (33.3%)
Immunotherapy None Steroids Intravenous immunoglobulins Steroids and plasma exchange Steroids and intravenous immunoglobulins Steroids and azathioprine Steroids and rituximab	14 (29.2%) 19 (39.6%) 4 (8.3%) 2 (4.2%) 6 (12.5%) 2 (4.2%) 1 (2.1%)
Final diagnosis Isolated myelitis Isolated optic neuritis NMOSD MOGAD Suspected autoimmune encephalitis NORSE Suspected autoimmune encephalopathy ADEM	8 (16.7%) 6 (12.7%) 3 (6.3%) 7 (14.6%) ** 17 (35.4%) 3 (6.3%) 3 (6.3%) 1 (2.1%)
Outcome at discharge (mRS)	1 (0–6)

Data expressed as number (percentage) or median (range) as appropriate. *mRS* modified Rankin Scale, *NMSOD* neuromyelitis optica spectrum disorder; MOGAD: Myelin oligodendrocyte glycoprotein (MOG) antibody-associated disorder, *NORSE* new-onset refractory status epilepticus; ADEM: acute disseminated encephalomyelitis

^*^Antibodies positivity:7 MOG (2 CSF restricted), 1 GFAP, 1 NMDAR, 1 AQP4, 1 amphyphisin, 1 titin

^**^Clinical features of MOGAD patients: 4 optic neuritis, 1 ADEM, 1 NMOSD, 1 encephalopathy

Clinical and paraclinical features of SARS-CoV-2-IgG seropositive patients (Table 2) revealed that only 9/16 patients reported an antecedent history of SARS-CoV-2 infection and 6 of them were symptomatic while 3 remained asymptomatic. Median time from SARS-CoV-2 infection to the onset of neurological symptoms was 31 days (range 3–45, *n* = 4). Among the 5 patients with demyelinating disorders and SARS-CoV-2-IgG positivity, two patients experienced isolated optic neuritis, two transverse myelitis, and one patient had a diagnosis of ADEM. One patient with transverse myelitis and the patient with ADEM had SARS-CoV-2-IgG CSF positivity. Among 23 patients with suspected encephalitis/encephalopathy and SARS-CoV-2-IgG positivity, 3 were diagnosed with encephalopathy, 1 with NORSE, 5 fulfilled diagnostic criteria for possible autoimmune encephalitis (in one case with anti-titin antibodies in both serum and CSF), and two patients fulfilled the diagnostic criteria for seronegative limbic encephalitis. Notably, one of the two patients with limbic encephalitis had CSF SARS-CoV-2-IgG positivity.

**Table 2:** Individual patient data of SARS-CoV-2 IgG-positive patients

Age, Sex	Serum/CSF SARS-CoV-2 IgG	History of SARS-CoV-2 infection/positive nasal swab	Symptomatic SARS-CoV-2 infection	Time from SARS-CoV-2 symptoms to neurological symptoms	MRI abnormalities	EEG abnormalities	CSF cells, CSF proteins	Diagnosis at discharge	Neurological antibodies positvity
53, M	±	No	No		Yes		2, 43	ON	None
58, F	+ /na	No	No					ON	None
75, M	±	Yes	No		No		2, 30	Encephalopathy	None
70, F	±	Yes	Yes	45	Yes	Yes	2, 27	Encephalitis	None
27, F	±	No	No				10, 25	Trasverse myelitis	None
73, F	±	Yes	Yes	3	Yes	Yes	4, 58	Encephalitis	Anti-titin in serum and CSF
70, F	±	No	No		Yes	Yes	1, 33	Encephalopathy	None
69, M	+ / +	Yes	Yes	37	Yes		1, 77	Encephalitis (limbic encephalitis)	None
77, M	±	Yes	No		No	Yes	1, 50	Encephalitis	None
61, M	+ / +	No	No		Yes		20, 35	Trasverse myelitis	None
20, F	±	No	No		No	Yes	2,30	NORSE	None
60, F	±	Yes	No		No	Yes	0, 21	Encephalitis	None
64, F	+ / +	Yes	Yes	25	Yes		22, 45	ADEM	None
71, F	±	Yes	Yes		No	Yes		Encephalopathy	None
58, M	±	No	No					Encephalitis	None
73, F	+ /na	Yes	Yes		Yes	Yes	16, 23	Encephalitis (limbic encephalitis)	None

The comparison between SARS-CoV-2-IgG seropositive and seronegative patients (Table 3) showed that seropositive patients were older (*p* = 0.006), more frequently with encephalopathy (*p* = 0.025), less frequently CSF pleocytosis (*p* = 0.043), a lower rate of neurological autoantibodies (*p* = 0.033), and were less likely to receive immunotherapy (*p* = 0.027). When comparing SARS-CoV-2-IgG seropositive and seronegative patients with atypical demyelinating disorders, no significant differences emerged (Supplemental Table 1). In patients with suspected autoimmune encephalitis/encephalopathy, SARS-CoV-2-IgG seropositive patients had fewer CSF cells and protein (*p* = 0.008) and trended towards less immunotherapy administration (*p* = 0.054) and more abnormal MRIs (*p* = 0.055, Supplemental Table 2).

**Table 3:** Comparison of clinical features according to SARS-CoV-2 serostatus

	Seropositive cases (*n* = 16)	Seronegative cases (*n* = 32)	*p*-value
Age	66.5 (20–77)	43 (5–83)	***0.006***
Female gender	10 (62.5%)	19 (59.4%)	0.54
Significant comorbidities	7 (43.8%)	8 (25%)	0.16
Seizures	5 (31.3%)	7 (21.9%)	0.356
Myelitis	3 (18.8%)	5 (15.6%)	0.541
Optic neuritis	3 (18.8%)	10 (31.3%)	0.288
Encephalopathy	12 (75%)	13 (40.6%)	***0.025***
Abnormal MRI	8 (50%)	25/31 (83.6%)	0.113
Abnormal EEG	8/8 (100%)	11/11 (100%)	n.a
CSF pleocytosis	4/13 (30.8%)	20/31 (65.4%)	***0.043***
CSF cells	3 (0–22)	9 (0–299)	0.186
CSF increased protein concentration	4/13 (30.8%)	17/31 (54.8%)	0.130
CSF protein concentration, mg/dL	35 (20.6–77)	56.3 (17–4395)	0.509
CSF restricted oligoclonal bands	0/8 (0%)	8/25 (32%)	*0.078*
Neurological autoantibodies positivity	1/16 (6.3%) *	11/32 (34.4%) **	***0.033***
Immunotherapy None Steroids Intravenous immunoglobulins Steroids and plasma exchange Steroids and intravenous immunoglobulins Steroids and azathioprine Steroids and rituximab	7 (43.8%) 4 (25%) 4 (25%) 0 1 (6.3%) 0 0	7 (21.9%) 15 (46.9%) 0 2 (6.3%) 5 (15.6%) 2 (6.3%) 1 (3.1%)	***0.027***
Final diagnosis Isolated myelitis Isolated optic neuritis NMOSD MOGAD*** Suspected autoimmune encephalitis NORSE Suspected autoimmune encephalopathy ADEM	2 (12.5%) 2 (12.5%) 0 0 7 (43.8%) 1 (6.3%) 3 (18.8%) 1 (6.3%)	6 (18.8%) 4 (12.5%) 3 (9.4%) 7 (21.9%) 10 (31.3%) 2 (6.3%) 0 0	*0.056*
Outcome at discharge (mRS)	3 (0–6)	1 (0–6)	0.556

Data expressed as number (percentage) or median (range) as appropriate. *mRS* modified Rankin Scale, *NMSOD* neuromyelitis optica spectrum disorder, *MOGAD* myelin oligodendrocyte glycoprotein (MOG) antibody-associated disorder, *NORSE* new-onset refractory status epilepticus, *ADEM* acute disseminated encephalomyelitis. P-values highligted in bold indicates statistically significant results, whereas p-values in italics indicates statistical trends.

^*^Antibodies positivity: 1 titin

^**^Antibodies positivity: 7 MOG (2 CSF restricted), 1 GFAP, 1 NMDAR, 1 AQP4, 1 amphyphisin

^***^Clinical features of MOGAD patients: 4 optic neuritis, 1 ADEM, 1 NMOSD, 1 encephalopathy

## Discussion

Herein, we describe a cohort of patients with neurological symptoms and concomitant SARS-CoV-2-IgG antibodies and compare them with clinically, geographically and time-matched SARS-CoV-2-IgG seronegative patients. We observed that (1) the rate of SARS-CoV-2-IgG positivity in patients referred for neurological autoantibody testing was 4%; (2) the rate of SARS-CoV-2 positivity was 33% in those patients who received a final diagnosis of atypical demyelinating disorders or autoimmune encephalitis/encephalopathy; (3) neurological symptoms also occur in patients with an unknown (44%) or asymptomatic antecedent infection (33%); (4) SARS-CoV-2-IgG seropositive cases showed less frequently antibody positivity and CSF inflammatory signs, despite the more common occurrence of encephalopathy; this difference was mainly due to cases with suspected encephalitis/encephalopathy; (5) SARS-CoV-2-IgG seropositive patients with atypical demyelinating disorders do not show clinical/paraclinical peculiarities.

An association between SARS-CoV-2 infection and inflammatory neurological disorders has been postulated, particularly in relation to Guillain-Barré syndrome, but remains unproven [9]. For other autoimmune neurological conditions, such as demyelinating disorders and autoimmune encephalitis, this relationship is largely based on case reports or small, often uncontrolled case series. Hence, our study has contributed to this literature by limiting a variety of biases with a robust control group. Our data suggest that demyelinating disorders may occur during/after SARS-CoV-2 infection, in accordance with the few isolated cases with myelitis or optic neuritis previously described [10, 11], and with a non-significant increase of MOG-IgG positivity in patients with positive SARS-CoV-2 IgG [12]. However, post-SARS-CoV-2 demyelinating attacks may not display a peculiar phenotype and this may relate to the pro-inflammatory cytokine milleu as a pathogenic agent which has been seen in other neurological conditions [13].

On the other hand, SARS-CoV-2-IgG-positive patients with suspected autoimmune encephalitis/encephalopathy have similar clinical features but different paraclinical findings in comparison with seronegative patients. Our results are in agreement with recent findings in terms of percentage of SARS-CoV-2-IgG seropositive cases (3 vs 4%) and negativity for neural antibodies [14]. The occurrence of encephalitis/encephalopathy in SARS-CoV-2-IgG seropositive patients might be linked, rather than with the presence of autoantibodies, with the cytokine storm associated with SARS-CoV-2 infection, where the release of pro-inflammatory cytokines can activate inflammatory processes and dysregulate immune responses [15]. In our cohort, we found low rates of SARS-CoV-2 antibodies in CSF samples, despite the high prevalence of encephalitis/encephalopathy. These results further support a predominant role of the cytokine storm rather than directly antibodies in the development of encephalitis/encephalopathy, as seen in patients developing Chimeric antigen receptor (CAR) T cell therapy-related toxicities [16].

Indeed, our observations are consistent with previous data reporting mostly negative antibody results in patients with encephalitis and acute SARS-CoV-2 infection and confirm the prominent role of cytokines rather than autoantibodies in SARS-CoV-2 related encephalitis [2, 14, 15].

Despite its limitations in terms of retrospective design, small size and possible referral bias, our study shows that CNS involvement can occur in patients with an antecedent SARS-CoV-2 infection, regardless of respiratory and systemic involvement, and usually do not display specific clinical manifestations or neuronal antibodies positivity. These observations retain relevant diagnostic and therapeutic implications for neurological symptoms occurring during and after the COVID-19 pandemic.

## Supplementary Information

Below is the link to the electronic supplementary material.

Supplementary file1 (DOCX 22 KB)

## Data Availability

Data are available upon reasonable request.
