# Supplementary material for: SARS-CoV-2 antibodies in inflammatory neurological conditions: a multicentre retrospective comparative study
Source: Immunol Res. 2023 May 12;71(5):717–24. doi: 10.1007/s12026-023-09384-2 (PMC10177711; doi:10.1007/s12026-023-09384-2)
Supplement: Supplementary file 1 — Supplementary file1 (DOCX 22 KB) [file 12026_2023_9384_MOESM1_ESM.docx]

Supplemental Table 1 – Comparison of patients with a final diagnosis of suspected atypical demyelinating disorder according to SARS-CoV-2 antibody status (n=25)

|  | Seropositive (n=5) | Seronegative (n=20) | *p-*value |
| --- | --- | --- | --- |
| Age | 58 (27-64) | 43 (5-74) | 0.133 |
| Female gender | 3 (60%) | 13 (65%) | 0.609 |
| Significant comorbidities | 3 (60%) | 4 (20%) | 1.113 |
| Seizures | 0 | 1 (5%) | 0.800 |
| Myelitis | 3 (60%) | 5 (25%) | 0.167 |
| Optic neuritis | 3 (60%) | 10 (50%) | 0.541 |
| Encephalopathy | 1 (20%) | 2 (10%) | 0.504 |
| Abnormal MRI | 3/3 (100%) | 16 (80%) | 0.547 |
| Abnormal EEG | 0 | 2/2 (100%) | n.a. |
| CSF pleocytosis | 3/4 (75%) | 9/19 (47.4%) | 0.329 |
| CSF cells | 15 (2-22) | 5 (0-69) | 0.281 |
| CSF increased protein concentration | 1/4 (25%) | 7/18 (36.8%) | 0.565 |
| CSF protein concentration, mg/dL | 39 (25-45.2) | 38.4 (17-4395) | 1.000 |
| CSF restricted oligoclonal bands | 0/4 | 5/18 (27.8%) | 0.325 |
| MOG/AQP4 autoantibodies positivity | 0 | 8 (40%) * | 0.116 |
| Immunotherapy  None  Steroids  Intravenous immunoglobulins  Steroids and plasma exchange  Steroids and intravenous immunoglobulins  Steroids and azathioprine  Steroids and rituximab | 2 (40%)  2 (40%)  0  0  1 (20%)  0  0 | 2 (10 %)  11 (55%)  0  2 (10%)  2 (10%)  2 (10%)  1 (5%) | 0.548 |
| Final diagnosis  Isolated myelitis  Isolated optic neuritis NMOSD  MOGAD**  Suspected autoimmune encephalitis  NORSE  Suspected autoimmune encephalopathy  ADEM | 2 (40%)  2 (40%)  0  0  0  0  0  1 (20%) | 6 (30%)  4 (20%)  3 (15%)  7 (35%)  0  0  0  0 | 0.121 |
| Outcome at discharge (mRS) | 2 (1-4) | 1 (0-5) | 1.000 |

Data expressed as number (percentage) or median (range) as appropriate.
mRS: modified Rankin Scale; NMSOD: neuromyelitis optica spectrum disorder; MOGAD: Myelin oligodendrocyte glycoprotein (MOG) antibody associated disorder; NORSE: new onset refractory status epilepticus; ADEM: acute disseminated encephalomyelitis
*Antibodies positivity: 7 MOG (2 CSF restricted), 1 AQP4.
** Clinical features of MOGAD patients: 4 optic neuritis, 1 ADEM, 1 NMOSD, 1 encephalopathy.

Supplemental Table 2 – Comparison of patients with a final diagnosis of suspected autoimmune encephalitis/encephalopathy according to SARS-CoV-2 antibody status (n=23)

|  | Seropositive (n=11) | Seronegative (n=12) | *p*-value |
| --- | --- | --- | --- |
| Age | 70 (20-70) | 47 (18-83) | 1.000 |
| Female gender | 7 (63.6%) | 6 (50%) | 0.407 |
| Significant comorbidities | 4 (36.4%) | 4 (33.3%) | 0.611 |
| Seizures | 5 (45.5%) | 6 (50%) | 0.579 |
| Myelitis | 0 | 0 | n.a. |
| Optic neuritis | 0 | 0 | n.a. |
| Encephalopathy | 11 (100%) | 11 (91.7%) | 0.522 |
| Abnormal MRI | 5/10 (50%) | 10/11 (90.9%) | *0.055* |
| Abnormal EEG | 8/8 (100%) | 9/9 (100%) | n.a. |
| CSF pleocytosis | 1/9 (11.1%) | 11 (91.7%) | ***<0.0001*** |
| CSF cells | 2 (0-16) | 20.5 (1-299) | ***0.008*** |
| CSF increased protein concentration | 3/9 (33.3%) | 10 (83.3%) | ***0.029*** |
| CSF protein concentration, mg/dL | 35 (20.6-77) | 309.5 (23-1932) | ***0.008*** |
| CSF restricted oligoclonal bands | 0/4 | 3/7 (42.9%) | 0.212 |
| Neurological autoantibodies positivity | 1 (9.1%) * | 3 (25%) ** | 0.329 |
| Immunotherapy  None  Steroids  Intravenous immunoglobulins  Steroids and plasma exchange  Steroids and intravenous immunoglobulins  Steroids and azathioprine  Steroids and rituximab | 5 (45.5%)  2 (18.2%)  4 (36.4%)  0  0  0  0 | 5 (41.7%)  4 (33.3%)  0  0  3 (25%)  0  0 | *0.054* |
| Final diagnosis  Isolated myelitis  Isolated optic neuritis NMOSD  MOGAD  Probable autoimmune encephalitis  NORSE  Suspected autoimmune encephalopathy  ADEM | 0  0  0  0  7 (63.6%)  1 (9.1%)  3 (27.3%)  0 | 0  0  0  0  10 (83.3%)  0  2 (16.7%)  0 | 0.148 |
| Outcome at discharge (mRS) | 5 (0-6) | 1 (0-6) | 0.280 |

Data expressed as number (percentage) or median (range) as appropriate.
mRS: modified Rankin Scale; NMSOD: neuromyelitis optica spectrum disorder; MOGAD: Myelin oligodendrocyte glycoprotein (MOG) antibody associated disorder; NORSE: new onset refractory status epilepticus; ADEM: acute disseminated encephalomyelitis
*Antibodies positivity: titin
** Antibodies positivity: 1 NMDAR, 1 GFAP, 1 amphyphisin
